# Does the sympathetic nervous system contribute to the pathophysiology of metabolic syndrome?

**DOI:** 10.3389/fphys.2015.00234

**Published:** 2015-08-25

**Authors:** Marina C. dos Santos Moreira, Izabella S. de Jesus Pinto, Aline A. Mourão, James O. Fajemiroye, Eduardo Colombari, Ângela A. da Silva Reis, André H. Freiria-Oliveira, Marcos L. Ferreira-Neto, Gustavo R. Pedrino

**Affiliations:** ^1^Department of Physiological Sciences, Center for Neuroscience and Cardiovascular Research, Federal University of GoiásGoiânia, Brazil; ^2^Laboratory of Pharmacology of Natural Products, Federal University of GoiásGoiânia, Brazil; ^3^Department of Physiology and Pathology, School of Dentistry, Universidade Estadual PaulistaAraraquara, Brazil; ^4^Department of Biochemistry and Molecular Biology, Federal University of GoiásGoiânia, Brazil; ^5^Laboratory of Experimental Physiology, Faculty of Physical Education, Federal University of UberlândiaUberlândia, Brazil

**Keywords:** obesity, insulin resistance, hypertension, cardiovascular diseases, central nervous system

## Abstract

The metabolic syndrome (MS), formally known as syndrome X, is a clustering of several risk factors such as obesity, hypertension, insulin resistance, and dislypidemia which could lead to the development of diabetes and cardiovascular diseases (CVD). The frequent changes in the definition and diagnostic criteria of MS are indications of the controversy and the challenges surrounding the understanding of this syndrome among researchers. Obesity and insulin resistance are leading risk factors of MS. Moreover, obesity and hypertension are closely associated to the increase and aggravation of oxidative stress. The recommended treatment of MS frequently involves change of lifestyles to prevent weight gain. MS is not only an important screening tool for the identification of individuals at high risk of CVD and diabetes but also an indicator of suitable treatment. As sympathetic disturbances and oxidative stress are often associated with obesity and hypertension, the present review summarizes the role of sympathetic nervous system and oxidative stress in the MS.

## Introduction

Cardiovascular diseases (CVD) are major causes of morbidity and mortality worldwide (WHO, 2013). The risk factors of CVD include hypertension, hypercholesterolemia, diabetes, genetic predisposition, obesity, sedentary lifestyle, and smoking (WHO, 2013). Unlike other risk factors, smoking and sedentary lifestyle are preventable. The risk factors that cannot be prevented often occur together. The clustering of these risk factors has been studied extensively (Kagota et al., 2010; Salazar et al., 2011; Gyawali et al., 2015). Reaven (1988) observed that insulin-resistant subjects often manifest obesity, high level of low-density lipoprotein (LDL)-cholesterol, low level of high-density lipoprotein (HDL)-cholesterol, fasting hyperglycemia and increased arterial blood pressure. These manifestations with multiple risk factors to the development of CVD were called Syndrome X (Reaven, 1988; Grundy et al., 2004). Later, the Syndrome X was renamed as Metabolic Syndrome (MS). In other words, MS is a clustering of several metabolic changes that affect the organism and often result in CVD.

The prevalence of CVD has increased since the advent of industrial revolution especially in western societies (Gottlieb et al., 2008). The less active lifestyle being promoted by modern transportation system, exclusion of manual jobs with high scale mechanized production and widespread consumption of fat rich food has contributed greatly to the increasing cases of obesity and MS. The consumption of industrial products which are also rich in sugar and salt elicits harmful effects on insulin resistance (Vidonho et al., 2004; Salazar et al., 2011) and blood pressure (Vidonho et al., 2004; He et al., 2008; Moreira et al., 2014). According to Stoian and Stoica (2014), MS patients exhibit lower concentrations of serum phosphate and magnesium as compared to subjects who did not meet the criteria for the diagnosis of this syndrome. Phosphate and magnesium are crucial to carbohydrate metabolism, thus, it is not surprising to suggest that lower level of these ions in MS patients can lead to a decrease in peripheral utilization of glucose as well as the development or aggravation of insulin resistance (Stoian and Stoica, 2014).

Some studies have demonstrated that oxidative and inflammatory stress play an important role in the initiation and progression of CVD (Toshima et al., 2000; Libby et al., 2002). It has been established that obesity could lead to an increase in the circulating markers of oxidative stress and inflammation (Festa et al., 2000; Keaney et al., 2003). As obesity is an important risk factor of MS, the establishment of a definitive relationship between inflammation and oxidative stress has attracted a lot of interest. However, there are still dearth of information in this regard.

In order to unravel the pathophysiology of MS, several definitions and diagnostic parameters have been proposed. The World Health Organization (WHO) defined MS as a group of risk factors of CVD and diabetes (Alberti and Zimmet, 1998; Grundy et al., 2004). WHO considered insulin resistance as the primary cause of MS (Grundy et al., 2004). This diagnostic consideration predicts diabetes in MS. However, the need of a specific test for insulin resistance makes MS diagnostic criteria by WHO less accessible (Grundy et al., 2004). According to WHO, the insulin resistance can be identified through one of the following factors: (i) type 2 diabetes; (ii) impaired fasting glucose; or (iii) impaired glucose intolerance. Besides the insulin resistance, at least two other risk factors are required to diagnose the syndrome (Alberti and Zimmet, 1998; Grundy et al., 2004). Some of the diagnostic criteria for MS are shown in Table 1.

**Table 1:** **WHO—Diagnostic criteria for metabolic syndrome**.

**Factor**	**Limit levels**
High blood pressure	≥140/≥90 mmHg or antihypertensive medication
Plasma triglycerides	≥150 mg/dL (1.7 mM)
HDL cholesterol	
Men	< 35 mg/dL (0.9 mM)
Women	< 39 mg/dL (1.0 mM)
Body mass index	>30 kg/m^2^
Urinary albumin excretion rate	≥20 μg/min

## Major components of metabolic syndrome

### Abdominal obesity

Obesity is defined by WHO as an abnormal or excessive fat accumulation that causes health problems (Mendis et al., 2015). The body mass index (BMI) is a parameter being are used to classify overweight and obesity in adults. BMI is calculated using the formula weight/height^2^ (kg/m^2^). WHO defines a BMI greater than or equal to 30 kg/m^2^ as obesity. Although the prevalence of obesity has been doubled worldwide since 1980, it is still a preventable disease. Nowadays, obesity kills more people than underweight globally. In 2014, it was reported that 39% of the adults (≥18 years) were overweight and 13% obese. About 42 million of people under 5 years of age were overweight in 2013 (Mendis et al., 2015).

Basically, obesity is caused by the imbalance between the calories consumed and calories expended. The higher consumption of calories as compared to the calories expended has being associated with weight gain. The accumulation of adipose tissue in the abdominal region causes abdominal obesity. Several studies have demonstrated that abdominal obesity which can be measured by the waist circumference has a strong correlation with the development of CVD (Ferreira and Moisés, 2000; Salazar et al., 2011; Traissac et al., 2015).

Considering the strong relationship among obesity, MS and cardiovascular risk factors, it is important to unravel the autonomic disturbances in obesity. Though the autonomic imbalance is not homogeneous in obesity, some studies demonstrated sympathetic hyperactivity in most part of obese individuals (Lopes and Egan, 2006). In the obese individuals, the sympathetic tone is increased in target organs such as kidney, skeletal muscle and peripheral vessels to elicit hypertension (Esler et al., 2001; Lopes and Egan, 2006).

### Hypertension

Hypertension is, by definition, a sustained increase in the arterial blood pressure (WHO, 2013). Nowadays, hypertension and its complications is one of the major cause of death worldwide. Factors such as increase in the sympathetic activity and dysregulation in sodium homeostasis could cause an increase in the arterial blood pressure. Although the pathogenesis of hypertension is still unclear, salt consumption has been implicated as one of the important causes of this disease (da Costa Lima et al., 1997; Ito et al., 1999; Contreras et al., 2000; Sacks et al., 2001; Rodriguez-Iturbe and Vaziri, 2007; He et al., 2008; Moreira et al., 2014). In addition, sedentary lifestyle, obesity and smoking are other factors that contribute to increase in blood pressure (Filho et al., 2008). The long-term increase in arterial blood pressure often affects some organs, like heart and kidneys. The higher the blood pressure, the greater is the resistance needed by heart to function. A higher blood pressure could lead to an increase in the frequency and contractile force. In the long-term, this changes in blood pressure could compromise cardiac function (Zile, 2002; Cingolani et al., 2003; Chen-Izu et al., 2007). According to WHO, hypertension is a systolic blood pressure equal to or above 140 mmHg and/or diastolic blood pressure equal to or above 90 mmHg (WHO, 2013).

Endothelial dysfunction has been identified in hypertension. Endothelium is a cellular layer on the vascular wall that controls the traffic of small and large molecules, and maintains the integrity of vascular wall (Carvalho et al., 2001). The endothelium controls the expansion and contraction of blood vessels by producing local mediators that are involved in vasodilation (endothelium-derived relaxing factors–EDRFs) or vasoconstrictor (endothelium-derived constriction factors–EDCFs) in response to changes in blood flow or vasoactive agents (Carvalho et al., 2001). The endothelium has multiple and important roles in physiological events: (i) it acts as hemodynamic sensor by receiving and transmitting signals from the extracellular matrix and cells; (ii) it produces mediators that interfere with growth, activity, migration and cell death; and (iii) it preserves the adaptive changes to circulatory requirements (Carvalho et al., 2001).

The principal EDRFs are nitric oxide (NO), the endothelium-derived hyperpolarizing factor (EDHF) and prostacyclin (PGI2). Angiotensin II (Ang II) and superoxide or reactive oxygen species (ROS) are among the main EDCFs (Kang, 2014). In pathophysiological situations, such as hypertension, an increase of EDCFs occurs. In this condition, study has shown an increase in the release of endothelial derived contractile factors cyclooxygenase (e.g., PGH 2) in response to acetylcholine and angiotensin II in aorta and mesenteric arteries of SHR (Côrtes et al., 1996). This result suggests endothelial dysfunction in hypertension. Under physiological conditions, there is a balance between the release, and production of the most important relaxing and contractile factors. This balance could be changed by attenuation of vasodilatory effect of endothelium. An apparent decrease in vascular endothelial-dependent relaxation is called endothelial dysfunction (Carvalho et al., 2001). The mechanisms involved in endothelial dysfunction as found in hypertension is multifactorial. Preclinical studies have shown an increase in the basal activity of nitric oxide synthase (NOS) and a decrease in the expression and activity of soluble guanylyl cyclase in smooth muscle of spontaneously hypertensive rats (Bauersachs et al., 1998; Kojda et al., 1998). On the other hand, in Dahl rats with salt-sensitive hypertension, there is a decrease in the responsiveness of vascular smooth muscle cells due to a decrease in the eNOS activity and NO production (Luscher and Vanhoutte, 1986; Hayakawa and Raij, 1998). EDHF has been described as one of the principal mediators of endothelium-dependent vasorelaxation in normotensive animals. The contribution of EDHF-mediated relaxation appears significantly greater in small resistance vessels than in large conduit vessels (Félétou and Vanhoutte, 2006). A reduction in the release of EDHF may lead to endothelial dysfunction and subsequently arterial hypertension (Fujii et al., 1992).

The oxygen and oxidative reactions in the body are vital for energy supply and defense against invaders. Under physiological conditions, the enzyme superoxide dismutase is responsible for preventing the formation of reactive species of oxygen such as peroxynitrite (ONOO-) which has a detrimental effects in the body (McIntyre et al., 1999). However, under oxidative stress condition as in MS, a large amount of O_2_ reacts with NO to form (ONOO–). This reaction could lead to a significant reduction in the bioavailability of endothelial NO.

Nitric oxide are highly relevant in endothelial dysfunction because in this pathology, production may be reduced due to changes in NOS3 (Nitro oxide synthase type 3) protein as observed in animal models of cardiovascular disease such as SHR, DOCA, diabetic rats (Hink et al., 2001; Sullivan et al., 2002). The reduction in the bioavailability of NO is considered as part of the mechanism for endothelial dysfunction in oxidative stress. NO could reacts with O_2_ to cause vasoconstriction, vascular damage and lipid peroxidation (Virdis et al., 2002).

In summary, endothelial dysfunction is characterized by an imbalance in the release of vasoconstrictors and the endothelium-dependent relaxants. Hence, increases in EDCFs are common in pathophysiological condition like hypertension.

### Insulin resistance

The insulin-resistance occurs when the body cells become less sensitive and resistant to insulin. Insulin, an hormone which facilitate glucose absorption, is produced in the beta cells of the pancreas (The IDF consensus worldwide definition of the MS: IDF, 2006). Once the glucose cannot be absorbed by the cells, it remains in the blood and subsequently triggers off the production of more insulin (hyperinsulinaemia reflex). The over production of insulin often wears off beta cells and diminishes its capacity to produce insulin. This condition generally leads to hyperglycaemia and type 2 diabetes (WHO and IDF, 2006; The IDF consensus worldwide definition of the MS: IDF, 2006). The insulin-resistance promotes damage to several insulin-sensitive organs such as liver and kidneys. The hyperinsulinaemia reflex increases the release of triglycerides by liver into the bloodstream and subsequent decrease in the level of HDL cholesterol, increase in the level of small and dense particles of LDL cholesterol (Reaven, 1988, 2003; Yoon et al., 2014). The hyperinsulinaemia reflex can contribute to the pathophysiology of the essential hypertension through an increase in renal water absorption and/or increase in the sympathetic activity (Reaven, 2003; Yoon et al., 2014). The insulin resistance is a major risk factor of type 2 diabetes and atherosclerotic complications such as coronary artery disease, stroke and peripheral arterial disease (Yoon et al., 2014).

### Dislypidemia

The dislypidemia is considered as an unbalance serum level of LDL (which increases) and HDL cholesterol particles (which decreases) (Reaven, 1988). As defined by Kaur (2014), dyslipidemia is characterized by spectrum of qualitative lipid abnormalities that reflect perturbations in the structure, metabolism, and biological activities of both atherogenic lipoproteins and antiatherogenic HDL cholesterol. Dislypidemia is frequently associated to insulin resistance. As mentioned above, the hyperinsulinaemia reflex, caused by the insulin resistance, promotes increase in the hepatic release of triglycerides to the blood. This release could decrease the level of HDL cholesterol and increase the level of small and denser particles of LDL cholesterol (Reaven, 1988, 2003; Yoon et al., 2014).

The increase in serum level of small and denser LDL cholesterol particles could lead to the accumulation of triglyceride in the vessels and development of atherosclerosis among other cardiovascular complications (Reaven, 1988, 2003).

### Inflammation and oxidative stress in metabolic syndrome

The increase in oxidative stress and inflammatory state are known to play an important role in the initiation and progression of CVD (Toshima et al., 2000; Libby et al., 2002). Obesity and MS have been associated with increased circulating markers of oxidative stress and inflammation (Festa et al., 2000; Keaney et al., 2003). However, there are still few information on the relationship between MS and inflammation/oxidative stress. The possible link between MS and inflammation is resistance to insulin (RI) (Volp et al., 2008). A defective insulin action in target tissues (muscle, liver, and adipose tissue) could increase chronic inflammation (Dandona et al., 2007). T cells elaborate inflammatory and anti-inflammatory properties of cytokines by stimulating macrophages, endothelial cells and smooth muscle cells (Volp et al., 2008). The key inflammatory cytokines markers include interleukin-6 (IL-6), tumor necrosis factor-α (TNF-α), interleukin-8 (IL-8), interleukin-1β (IL-1β), CD40, CD40L, and C-reactive protein (Kon et al., 2005; Wu and Wu, 2006). Since the adipocytes cells are the main producers of cytokines proinflammatory, there is a strong relationship between increased secretion and higher levels of cytokines in obese people. This phenomenon increases the risk of developing MS (Vanhala et al., 2006).

IL-6 is a pro-inflammatory cytokine involved in the development of hyperinsulinemia and MS. This cytokine increases lipolysis to release free fatty acids and glycerol while reducing the expression of insulin receptor substrate-1 (IRS-1) and GLUT4 in muscle and liver tissues (Francisco et al., 2006). Though IL-6 is mainly secreted by adipocytes, it is produced by smooth muscle cells, endothelial cells, monocytes and macrophages and may contribute to the development of atherosclerotic lesions through its paracrine, autocrine, and endocrine effect. Studies have correlated the values of serum IL-6 with the waist circumference (Rexrode et al., 2003). People with central obesity are at increased risk to develop MS.

The TNF-α is a cytokine with autocrine, paracrine and endocrine action (Ruan and Lodish, 2003). In obese humans, there is an inverse correlation between TNF-α and glucose metabolism (Winkler et al., 2003). Insulin signaling suppression by the TNF-α reduces the translocation of glucose transporter (GLUT-4) to the membrane and consequently promotes a decrease in insulin-mediated glucose uptake by cells. It is also known that the expression of mRNA and TNF-α secretion are higher in obese person (Hsueh and Law, 2003).

IL-1β is responsible for increase in the expression of endothelial adhesion molecules which facilitate the aggregation of other inflammatory cells in the activated endothelium (Francisco et al., 2006). IL-1β, together with TNF-α, stimulates IL-6 production by smooth muscle cells and increases the expression of macrophages. This process is associated with the progression of inflammatory and atherosclerosis processes (Francisco et al., 2006). IL-18 is a proinflammatory cytokine with pleiotropic action which induces the secretion of the cytokines IL-6, TNF-α, IL-1β and endothelial adhesion molecules (Francisco et al., 2006). This cytokine exerts chemotaxis of human T cells to promote the recruitment into the atherosclerotic plaque. It has been proposed that the IL-18 induces the expression of several matrix metalloproteinases, which may weaken the fibrous cap of injury atherosclerotic (Hung et al., 2005).

The cytokines CD40 and CD40L are expressed by macrophages, lymphocytes T, platelets, endothelial cells and muscle cells flat (Hung et al., 2005). The system CD40/CD40L exerts various pro-inflammatory and pro-thrombotic effects by: (i) stimulating the production of endothelial cells free radicals to antagonize nitric oxide production; (ii) inducing expression of endothelial cells and smooth muscle adhesion molecules; (iii) stimulating the expression of proinflammatory cytokines and chemokines; (iv) inducing tissue factor expression on endothelial and smooth muscle cells to promote an increase in potential thrombogenic plate; and (v) participating in the activation of platelet (Angelico et al., 2006). Data has shown that CD40 which is expressed on the surface of platelets could activate platelet to promote thrombus formation (Angelico et al., 2006).

C-Reactive Protein (CRP) is synthesized by the liver and regulated by cytokines, predominantly IL-6, TNF-α, and IL-1 (Abdellaoui and Al-Khaffaf, 2007). Its levels are increased during acute inflammatory process. Mild increase in the level of CRP also occurs in chronic inflammatory condition such as atherosclerosis. The levels of this protein almost triple in the presence of peripheral vascular disease risk (Abdellaoui and Al-Khaffaf, 2007). It has been shown that MS patients have CRP serum levels significantly higher than people without MS (Bahia et al., 2006).

Macrophages are a heterogeneous population of immune cells that have a range of roles in both the induction and resolution of inflammation (Dey et al., 2015). The pleiotropic responses are coordinated through distinct programs of macrophage activation classified as classical (or M1) and alternative (or M2) activation (Gordon, 2003; Martinez et al., 2008).

It has been shown that the immune response that is activated during inflammation and obesity-induced insulin resistance has the same M1 mechanism (Takeda et al., 2003). In classical activation of macrophages, lipids derived from bacteria bind to Toll-like receptor 4 (TLR4) and activate signaling pathways that induce, for example, the release of NF-kB inflammatory molecules (TNF, IL-6 and 2) (Takeda et al., 2003). In obesity and inflammation, saturated fatty acids activate TLR4 to promote inflammatory responses (Shi et al., 2006; Kim et al., 2007; Nguyen et al., 2007). In fact, acute infusion of lipids into mice potentiates the insulin resistance in both adipose tissue and skeletal muscle in a TLR4-dependent manner (Kim et al., 2007). In addition, other studies have shown that white adipose tissue of obese rats have mainly macrophages with classically activated—M1 phenotype (Bouloumié et al., 2005; Ferrante, 2007). Alternatively, during the resolution of inflammation, the balance of macrophage activation toward an M2 phenotype occurs in order to promote clearance of debris and inhibit the production of inflammatory mediators to restore tissue homeostasis (Mills et al., 2014). M2 macrophages produce anti-inflammatory cytokines and express endocytic receptors. These cells promote the clearance of apoptotic cells, proliferation and wound healing (Mills et al., 2014). Together, these data suggest a model in which increased flux of saturated fatty acids, as seen in obese states stimulates classical macrophage activation, tissue inflammation, and insulin resistance.

Although the number of classically activated adipose tissue macrophages increases with obesity, adipose tissue of lean animals contains a moderate number of macrophages. The adipose tissue macrophage of lean mice under non-inflammatory conditions express high levels of substances encoded by genes of alternatively activated M2 macrophages that promotes tissue homeostasis and repair (Hung et al., 2005; Vanhala et al., 2006). As suggested by Palaniappan et al. (2003), obesity results in a change in the activation pathway of macrophages.

Van Guilder et al. (2006) evaluated a possible synergistic effect of MS and obesity on the circulating markers of oxidative stress and inflammation. In that study, the authors observed that MS heightens oxidative stress and inflammatory burden in obese adults, thereby suggesting that MS and obesity have a synergistic effect on oxidative stress and inflammation markers (Van Guilder et al., 2006). Furthermore, the authors suggested that the increased oxidative and inflammatory stress may contribute to risk of coronary heart disease and cerebrovascular disease in obese adults with MS (Van Guilder et al., 2006).

Several studies have related the adipose tissue to the elevated markers of oxidative stress and inflammation once the expression and secretion of such markers increase in proportion to adiposity (Mohamed-Ali et al., 1998; Bertin et al., 2000; Kern et al., 2001; Van Guilder et al., 2006). However, as observed by Van Guilder et al. (2006), the adiposity alone do not seems to be the primary cause of oxidative stress and inflammation once the markers are increased only in the obese individuals diagnosed with MS.

In addition, an increase in glucose metabolism has been implicated in oxidative stress—MS, relation (Furukawa et al., 2004; Tangvarasittichai, 2015). During the metabolism of glucose (glycolysis and tricarboxylic acid—TCA cycle), the electron donors NADH (nicotinamide adenine dinucleotide) and FADH2 (flavin adenine dinucleotide) are generated (Tangvarasittichai, 2015). In the case of over nutrition or obesity, a large amount of glucose is oxidized in such a way that an increase in the generation of NADH and FADH2 in the mitocondrial electron transport chain subsequently result in an increase in superoxide generation (Tangvarasittichai, 2015). Furthermore, the increase in free fatty acid and acetyl coenzyme A (CoA) oxidation (due to the excessive of free fatty acids) in TCA cycle generate more molecules of NADH and FADH2 to be oxidized, and overproduction ROS (Furukawa et al., 2004; Tangvarasittichai, 2015). Moreover, NADPH oxidase is involved in fatty acids—ROS generation in adipocytes once the treatment with NADPH oxidase inhibitor blocks the ROS generation (Furukawa et al., 2004; Tangvarasittichai, 2015).

Like oxidative stress, inflammatory state is also an important feature of MS. Inflammation is one the manifestations of oxidative stress (Roebuck, 1999; Tangvarasittichai, 2015). Festa et al. (2000) demonstrated that chronic subclinical inflammation is a key feature of the MS and components of MS (dyslipidemia, abdominal obesity, and hypertension) increase in parallel to the increasing levels of plasma CRP in non-diabetic individuals. It is possible that chronic inflammation triggers MS (Festa et al., 2000), overnutrition, cytokine hypersecretion, insulin resistance, and diabetes in predisposed individuals (Festa et al., 2000). Festa et al. (2000) also suggested that the decrease in insulin sensitivity may lead to increase in CRP expression by counteracting the physiological effect of insulin on hepatic protein synthesis (Campos and Baumann, 1992; Festa et al., 2000).

Although there are many studies focusing on oxidative stress, inflammation and MS (Campos and Baumann, 1992; Roebuck, 1999; Festa et al., 2000; Furukawa et al., 2004; Tangvarasittichai, 2015), there is still no clear consensus about the causal relationship among the triad oxidative stress—inflammation—MS.

## The sympathetic nervous system and the metabolic syndrome

The sympathetic nervous system (SNS) is an arm of the autonomic nervous system which plays vital role in the regulatory mechanisms of blood pressure, sodium balance and maintenance of homeostatic state. The SNS is fundamental in the control of daily energy expenditure through the regulation of resting metabolic rate and thermogenesis in response to physiological stimuli, changing energy states, food intake, carbohydrate consumption and hyperinsulinemia (Thorp and Schlaich, 2015). Furthermore, the activation of sympathetic nerves in target organs like liver, pancreas, skeletal muscle, and adipose tissue can elicit acute catabolic responses (i.e., glycogenolysis and lipolysis) (Thorp and Schlaich, 2015).

Over activation of SNS is strongly associated with two components of the MS, i.e., obesity and hypertension (Tentolouris et al., 2006). In fact, enhanced SNS activation exerts unfavorable effects like cardiac hypertrophy, arterial remodeling, and endothelial dysfunction on the cardiovascular system (Grassi and Seravalle, 2006). Increase in sympathetic activity enhances systemic and regional norepinephrine spillover and elevate resting heart rate. This condition has been linked to hypertension, obesity, and insulin resistance (Mancia et al., 2007). Furthermore, it has been shown that high levels of fasting insulin, an index of insulin resistance, were positively associated with the low-to-high frequency (LF/HF) ratio of the heart rate variability (HRV)—an index of the sympathovagal balance at the heart level (Emdin et al., 2001).

In view of the strong relationship among obesity, MS and the development of cardiovascular risk factors, it is important to elucidate autonomic disturbances that occur in obese individuals. As cited by Hall et al. (2000), there are two lines of evidences about the central nervous system involvement in obesity-induced hypertension: (i) increase in sympathetic activity in obese as compared to lean subjects; and (ii) attenuation of obesity-related hypertension through pharmacological blockade of adrenergic activity (Landsberg and Krieger, 1989; Grassi et al., 1995; Hall et al., 2000). Though the autonomic disorders are not homogeneous in obesity, some studies have demonstrated that most individuals exhibit sympathetic hyperactivity (Lopes and Egan, 2006). Both baroreflex sensitivity (BRS) and impaired HRV in obese women (Skrapari et al., 2007). Esler et al. (2001) demonstrated that the sympathetic tone in obese individuals is increase in some target organs like kidney, skeletal muscle and vessels. Sympathetic hyperactivity in obesity indicates that obesity impairs renal-pressure natriuresis, increases renal tubular sodium reabsorption and causes hypertension (Kassab et al., 1995; Hall et al., 2000).

It is known that sympathetic disturbances are directly related to increase in arterial blood pressure (Tan et al., 2010; Oliveira-Sales et al., 2014). In humans, several features confirm typical increase in sympathetic tone as observed in obesity. Various studies have demonstrated increased blood pressure and serum catecholamine levels in obese individuals. The loss of weight is associated to the decrease in plasma concentration norepinephrine (Tuck, 1992; Lopes and Egan, 2006). Obese hypertensive children show increase in sympathetic nerve activity (Rocchini et al., 1989; Lopes and Egan, 2006). However, in these patients, a low salt diet (or hyposodic diet) is capable of promoting a decrease in arterial pressure (Rocchini et al., 1989; Lopes and Egan, 2006). These studies point out the fact that sympathetic hyperactivity is related to sodium retention and increase of arterial blood pressure in obese children (Lopes and Egan, 2006). Furthermore, muscular sympathetic nerve activity (MSNA) is increased in obese (normotensive and hypertensive) as compared to non-obese normotensive individuals (Grassi et al., 2000). Moreover, it has been shown that the MSNA and the plasmatic norepinephrine are reduced and the BRS is increased after weight loss in normotensive obese individuals (Grassi et al., 1998; Lopes and Egan, 2006). The SNS response is blunted in obese subjects despite the fact that plasma insulin levels were almost 45% higher in the obese as compared to the lean patients (Tentolouris et al., 2003).

The heart rate variations are a visible effect of the autonomic influences on the heart in cases of emotional stress. An inability to sustain varying heart rate is an important risk factor to the development of CVD (Hemingway et al., 2001; Brunner et al., 2002). The study of Brunner et al. (2002) demonstrated a relative sympathetic dominance and a lower vagal tone to the heart in MS cases, thereby indicating unbalance sympathovagal in those individuals. Jamerson et al. (1993) demonstrated an inverse relationship between sympathetic vascular tone and the insulin-mediated cellular consumption of glucose. Thus, it is not surprising to speculate that the increased SNA as observed in obese individuals increases the vascular constriction and impairs the glucose transportation into the cells (Grassi et al., 2000; Lopes and Egan, 2006). Previous studies with obese models have implicated vascular constriction in insulin-resistance (Laakso et al., 1990; Lopes and Egan, 2006). Specific alpha-adrenergic vasoconstriction seems to be more malefic on glucose consumption than the angiotensin-induced vasoconstriction (Jamerson et al., 1993; Lopes and Egan, 2006). This assumption suggests, once again, sympathetic influence on glucose metabolism.

In patients with type 2 diabetes mellitus (T2DM), MS has approximately 70% of prevalence rate (Monami et al., 2007; Bianchi et al., 2008). The basic mechanism involved in the pathogenesis of T2DM is the insulin resistance. The insulin resistance, in turn, is strongly associated with sympathovagal imbalance. Furthermore, many data suggest the involvement of increased SNS activity in insulin resistance (Mancia et al., 2007). Epidemiological studies have found a correlation between insulin resistance and hypertension (Modan and Halkin, 1991; Skyler et al., 1995). In patients with type 1 diabetes mellitus (T1DM), the hypertension is usually developed after the onset of nephropathy and it is associated with rennin-angiotensin induced SNS activation (Perin et al., 2001). In contrary, the prevalence of hypertension in patients with T2DM is extremely common (Perin et al., 2001). Thus, it can be assumed that Insulin resistance and hypertension as observed in the MS are closely linked with sympathetic overactivation (Frontoni et al., 2005).

The SNS activity plays a crucial role in the regulation of circulation and blood pressure (Fisher and Paton, 2011; Zubcevic et al., 2011). Sympathetic vasomotor and cardiac neural activities are induced by the sympathetic preganglionic neurons in the spinal cord. These neurons receive tonic excitatory drive from pre-sympathetic networks within the brainstem and hypothalamus (Dampney, 1994; Guyenet et al., 1996; Dampney et al., 2002; Madden and Sved, 2003). Increased in SNS activity has been linked to the pathogenesis of hypertension in humans with essential hypertension (Abboud, 1984; Mancia et al., 1999; Esler et al., 2001; Guyenet, 2006). Sympathetic overload is implicated in the pathogenesis and/or deterioration of essential hypertension through the modification of heart rate, cardiac output, peripheral vascular resistance and renal sodium retention (Grassi and Seravalle, 2006). The study of sympathetic nerve firing rate in hypertensive patients has shown sympathetic overactivity in young, middle-aged, and elderly hypertensive (Grassi et al., 2000). Some studies with essential hypertensive patients have plasmatic overflow of norepinephrine. This overflow indicates an increase in the activation of sympathetic outflow to the heart, kidneys and cerebrovascular circulation of these individuals (Esler et al., 1991; Mancia et al., 2007). These observations are evidences that some target organs are negatively affected by increased blood pressure (Grassi et al., 2007). Moreover, increase in heart rate has been observed in subjects with MS as compared to those without MS (Mancia et al., 2007). In this way, the physical inactivity can be associated with obesity as well as to the increase in cardiac sympathetic drive in the MS.

The SNS disturbances are closely related to all the main features of the MS. Although SNS participation in the pathophysiology of MS is clear, it is difficult to determine whether the metabolic changes are responsible for sympathetic disturbances or vice versa.

## Role of leptin in the elevation of sympathetic activity

Obesity, an important risk factor for the development of MS, is characterized by the increase in size and number of adipocytes (cells that produce adipokines, Martínez-Martínez et al., 2014). Leptin is one of the adipokines that has been postulated as a link between obesity and cardiovascular damage (Martínez-Martínez et al., 2014). Leptin is a peptide synthesized and secreted from white adipose tissue (Head et al., 2014) in addition to other sources like placenta, stomach, and heart (Zeidan et al., 2011). Leptin expression can be induced by obesity, insulin and TNF-α. This adipokine has been implicated in numerous physiological functions such as immune response and reproduction (Frühbeck, 2002; Mark, 2013). However, its main action is related to glucose homeostasis and regulation of appetite. This hormone provides feedback to the CNS on the status of peripheral energy reserves (Rahmouni, 2010).

Plasma leptin concentrations are significantly elevated in several rodent and human models of obesity in a proportional manner to adiposity (Magni et al., 2000). Although circulating levels of leptin rise in obesity, these individuals are thought to be leptin resistant due to lack of satiation (Magni et al., 2000). It is known that systemic infusion of leptin increased renal sympathetic nerve activity (RSNA) and supplies (Haynes et al., 1997). Central infusion of this peptide increases blood pressure (Shek et al., 1998). Head et al. (2014) recently demonstrated that central infusion of leptin antagonist in obese rabbits is able to return blood pressure to their basal levels (Head et al., 2014). These findings may contribute to understand an obesity-induced hypertension. Furthermore, recent studies have demonstrated that antagonism of central leptin receptor, LepR, caused a reduction in BP and HR in hypertensive mice (Tumer et al., 2007). The cardiovascular effects of leptin administration are not exclusively due to its central action. In fact, it has been shown that systemically administration of leptin antibodies elicited similar changes as compared to central administration (Tumer et al., 2007).

In an animal model of non-obese animals with hyperleptinemia, leptin increased systolic blood pressure and promoted an increase in intrarenal and systemic oxidative stress (Beltowski et al., 2004). In these model, the increase in ROS cause inactivation of nitric oxide. This effect can explain the leptin-associated hypertension in this model (Beltowski et al., 2004; Martínez-Martínez et al., 2014). Renal sodium retention has also been implicated in hyperleptinemia hypertension (Martin et al., 2008). Beltowski et al. (2004) have demonstrated that hyperleptinemia decreases urinary sodium excretion to promote volume retention and consequent increase in arterial blood pressure. Other harmful effects such as atherosclerosis (Gruen et al., 2007), inflammation, thrombosis and cardiac myocyte hypertrophy (Northcott et al., 2012) have been associated with leptin. Based on these observations, it is reasonable to suggest that pharmacological approaches targeting leptin's effects could represent a potentially useful therapeutic strategy for the treatment of obesity-associated hypertension among other cardiovascular disease.

## Treatment of metabolic syndrome

Since MS is a clustering of dysfunctions, different treatment strategy has been adopted. WHO suggested treatments focusing on insulin resistance as first-line therapy. A more active lifestyle (which could lead to the weight loss) could be a promising therapy as it decreases insulin resistance. Since there are direct relationships between sedentary lifestyle and cardiovascular risk factors, the benefits of physical exercises on the prognosis of the MS are clear (Lakka et al., 2003; Rennie et al., 2003). In fact, translational studies demonstrate lower insulin levels and increased insulin sensitivity in athletes as compared to match-aged sedentary individuals (Nuutila et al., 1994; Roberts et al., 2013). Eriksson et al. (1997) demonstrated that one single session of exercise increase the glucose offer mediated by insulin in healthy and insulin resistant obese and type 2 diabetes individuals. Chronic exercise improves insulin sensibility in healthy, non-diabetic obese, type 1 and 2 diabetic individuals (Eriksson et al., 1997). Also, the use of drugs like metformin and thiazolidinediones (insulin sensitizers) have also been prescribed for insulin resistance treatment (Einhorn et al., 2003; Grundy et al., 2004).

As previously mentioned, the serum phosphate and magnesium is reduced in MS patients (Stoian and Stoica, 2014). The compensatory hyperinsulinemia can promote decrease of serum phosphate and magnesium, a vicious cycle which contributes to the pathophysiology of MS (Stoian and Stoica, 2014). Hence, a pharmacological treatment that restores the level of these ions could be beneficial (Stoian and Stoica, 2014) to MS patients.

## Final considerations

**Figure 1:**
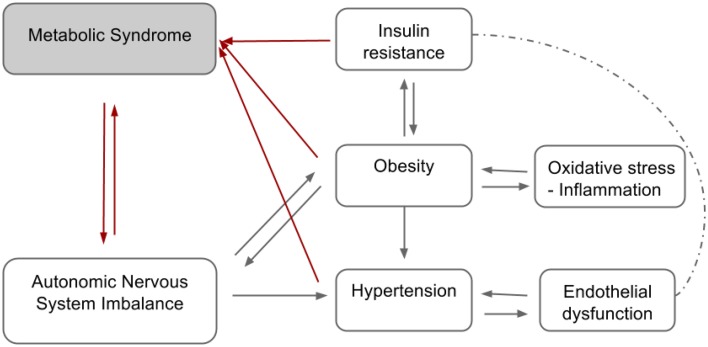
**Hypothetical diagram summarizing the relationship between the components of the metabolic syndrome**.

The definition and diagnostic criteria of MS are still controversial for obvious reasons. The divergent opinions on the diagnosis of MS among health institutions constitute challenges to its treatment and identification of individuals at high risk of CVD and diabetes. The diagram presented in the Figure 1 below summarizes the complex relationship between some of the MS components.

A clinical diagnosis of MS is critical as it affects therapeutic strategy. A multidisciplinary approach including lifestyle changes, pharmacological and surgical approaches could be helpful toward the management of this syndrome. Physical inactivity, insulin resistance, advance age, hormonal factors (androgens and corticosteroids), and diets rich in fats which promote abdominal obesity or adiposity have been consistently identified as major risk factors of MS. Atherogenic dyslipidemia, elevated blood pressure, smoking, elevated glucose, prothrombotic, and proinflammatory state are cardiovascular risk factors that accompany MS. Change in lifestyle and anti-obesity drugs among others could engender effective prevention or treatment of MS. Although, lifestyle changes remain first-line therapy for the improvement of all the underlying metabolic risk factors, cases of unsuccessful lifestyle modification therapy can be substituted with anti-obesity treatment. Lifestyle therapy could dampen MS progression at every stage. This kind of therapy does not treat each risk factor in isolation but rather target multiple risk factors simultaneously. Although lifestyle therapy may not modify any given risk factor as much as drug, its benefit lies in the fact that it produces moderate reduction in all metabolic risk factors. In most situations, drug therapies might be required in the case of worsening condition of MS. The effectiveness of drugs targeting individual risk components of MS separately is still uncertain. This approach could lead to aggressive use of medications at the expense of lifestyle therapy. The ineffectiveness of some available drugs for the treatment of MS has been compounded by the impractical approach of simultaneous prescription and administration of all the drugs that could modify all of the risk factors. Current efforts being made to combine drugs into a single capsule that targets multiple risk factors seems ingenious as it could reduce the burden of polypharmacy. A single drug that can affect multiple metabolic risk factors simultaneously and provide health benefit to MS patient seems promising. Drugs that act as angiotensin and adrenergic (alpha and beta) receptors blockers and peroxisome proliferator–activator receptor-γ agonism could lower blood pressure, vascular and cardiac sympathetic tone as well as plasmatic glucose simultaneously. Though new drug development programme looks interesting, better understanding of MS, improved diagnostic criteria and treatment strategy is key to future clinical practice.

### Conflict of interest statement

The authors declare that the research was conducted in the absence of any commercial or financial relationships that could be construed as a potential conflict of interest.
